# Development and Preliminary Validation of ORCA-PD, an Online Rapid Cognitive Assessment for Parkinson Disease: Mixed Methods Study

**DOI:** 10.2196/90057

**Published:** 2026-06-08

**Authors:** Avigail Lithwick Algon, Sarah Brisman, Chi-Ying R Lin, William Saban

**Affiliations:** 1 Department of Occupational Therapy, Gray Faculty of Medical & Health Sciences, Sagol School of Neuroscience, Center for Accessible Neuropsychology, Tel Aviv University, Chaim Levanon St 55, Tel Aviv, 6139001, Israel, 1 0546428833; 2 Department of Neurology, Baylor College of Medicine, Houston, TX, United States

**Keywords:** online, neuropsychological testing, Parkinson disease, accessibility, cognition

## Abstract

**Background:**

Traditional in-person neuropsychological tests for Parkinson disease (PD) lack accessibility, scalability, and PD specificity. Mobility impairments hinder access to in-person assessments, and long waiting times for expert evaluation limit scalability. Common tools for cognitive screening, such as the Montreal Cognitive Assessment, are generic and not specific to PD.

**Objective:**

The goal of this study was to address these challenges by leveraging the internet. This study aimed to develop a sensitive tool to detect cognitive impairments in early- to mid-stage PD in an accessible and scalable manner.

**Methods:**

We developed the Online Rapid Cognitive Assessment for Parkinson’s Disease (ORCA-PD), a brief (~15 min), fully self-administered tool, tailored to detect PD-specific impairments remotely. In a cross-sectional study, 112 participants from diverse geographical locations completed the ORCA-PD and a usability questionnaire. Task selection for ORCA-PD was guided by meta-analyses and comprehensive reviews, which demonstrated medium-to-high sensitivity to PD compared to healthy controls.

**Results:**

Participants from more than 30 geographical locations showed a 93% (135/145) completion rate, with median usability ratings of 4 to 5 (IQRs 0-1) out of 5, indicating strong usability across a diverse sample. The ORCA-PD score significantly correlated with the Montreal Cognitive Assessment score (ρ=0.45, 95% CI 0.21-0.64; *P*<.001; n=56), supporting convergent validity. The ORCA-PD scores also followed expected significant trends: neurotypical controls scored higher than participants with PD (β=4.18; *P*=.048) and participants with PD without mild cognitive impairment scored higher than participants with PD with mild cognitive impairment (β=7.22; *P*=.046), confirming 2 discriminative abilities.

**Conclusions:**

ORCA-PD demonstrated usability and preliminary validity. ORCA-PD offers an accessible, scalable, and PD-sensitive cognitive screening test that could complement traditional in-person, supervised tools.

## Introduction

As global life expectancy increases, the disease burden of age-associated neurodegenerative diseases, such as Parkinson disease (PD), is rising [1]. PD is the most common age-related motor neurodegenerative disorder [2], characterized primarily by motor symptoms [3]. However, PD also leads to nonmotor symptoms, such as a decline in cognition [4]. This decline often remains covert and goes unnoticed by clinicians and people with PD [4]. Despite the importance of cognitive evaluations for people with PD, current in-person assessment tools present several challenges.

Traditional cognitive screening tools for PD have long faced challenges related to accessibility, scalability, and PD specificity [5]. Accessibility is a persistent challenge, particularly for people with PD who face barriers to in-person cognitive assessments [5-7]. It is often challenging for people with PD to attend in-person cognitive assessments, especially those with severe motor and mobility impairments [8]. Motor impairments, including bradykinesia and postural instability, can make travel particularly challenging. In addition, PD-related fatigue may reduce their capacity to participate in testing [9,10]. Cognitive deficits may further complicate participation, as people with PD may struggle with task switching [11], working memory [12], visuospatial abilities [13], and impulsivity [14]. Thus, people with PD often find it challenging to navigate and commute to laboratories and clinics.

Beyond accessibility concerns, scalability is limited by the fact that most cognitive assessments must be administered by trained professionals, such as neuropsychologists or neurologists, who are often in high demand [15]. This issue is especially pronounced for people with PD living in remote areas with limited specialist access [5]. Although telehealth approaches can extend clinician-led cognitive assessments to remote settings, they remain constrained by specialist availability, scheduling burdens, and clinician time, limiting their scalability for large-scale screening [16]. Furthermore, recruitment challenges often result in small sample sizes (frequently fewer than 30 participants), drawn from single institutions and limited geographic regions [5], raising concerns about sampling bias and the generalizability of findings [17].

Regarding specificity, commonly used cognitive screening tools such as the Montreal Cognitive Assessment (MoCA) [18] and the Mini-Mental State Examination(S) [19] are generic and not tailored to the cognitive profile of PD. Although tasks such as clock drawing and cube copying are intended to measure visuospatial and executive functions—both relevant to cognitive decline in PD—their reliance on motor execution may confound interpretation, since motor impairments can limit performance independently of cognitive status.

We propose using a web-based service to overcome these barriers. Recent work has explored the feasibility of web-based cognitive assessments to enhance accessibility and scalability. Behavioral researchers in different domains have been relying on the internet to reach larger [20] and more diverse populations [21]. Although self-administered cognitive tasks have been evaluated in neurotypical controls (NT) [22-25] and individuals with mild cognitive impairment (MCI) [26], relatively few studies have examined their usability and validity in PD [27-31]. Given the cognitive profile of PD [32] even before PD-dementia, it is important to use sensitive tools capable of detecting cognitive differences between PD and NT, as well as between participants with PD without MCI (PD-non-MCI) and participants with PD with MCI (PD-MCI). Thus, aiming to further address these accessibility, scalability, and PD-specificity challenges, we developed the Online Rapid Cognitive Assessment for Parkinson’s Disease (ORCA-PD), a fully self-administered, web-based cognitive screening tool. ORCA-PD was tailored to capture early- to mid-stage PD-specific cognitive impairments without increasing motor demands for in-person visits.

Here, we aimed to evaluate the feasibility and preliminary validity of ORCA-PD as a remote, self-administered cognitive screening tool tailored for PD. First, we evaluated whether ORCA-PD can be feasibly completed outside the clinic in diverse geographical locations and whether users find it usable. Second, to situate ORCA-PD within existing assessment practices, we examined its convergent validity with the MoCA. Finally, we tested its ability to distinguish neurotypical individuals from people with PD, as well as PD-non-MCI from PD-MCI cohorts.

## Methods

### Overview

First, we developed the ORCA-PD by selecting measures based on prior meta-analyses and reviews reporting medium-to-large effect sizes for differences between PD and NT. Second, to evaluate feasibility, we administered the ORCA-PD to people with PD and NT with or without MCI. Third, we tested whether performance declined as task difficulty increased, assessing the online tasks’ internal structure. Fourth, we administered a usability questionnaire.

### Participants: Power Analysis

To determine the required sample sizes, we performed a power analysis (α=.05; power=0.99) using effect sizes obtained from 5 in-person studies comparing PD and NT on the MoCA [33-37]. These analyses indicated a minimum required sample size of 14 participants per group. Accordingly, our larger sample size (>50 participants, as given below) provided sufficient power to detect group differences and accounted for the increased variability associated with remote administration (eg, differences in test conditions).

In this cross-sectional study, 112 participants (56 PD and 56 NT) completed the ORCA-PD. The groups were matched for age (*t*_107.06_=−1.71; *P=.*09) and gender (*χ*²_1_=0.00; *P*>.99). However, the PD group had significantly more years of education than the NT group (*t*_99.30_=−2.92; *P=.*004); thus, education was a covariate in the analyses. Table 1 shows the demographic and clinical characteristics of both groups. Within the sample, a subgroup of 56 participants (PD=41; NT=15) also completed the MoCA. Participants completed the MoCA during a videoconference session, while the ORCA-PD was sent to the participants to be completed on their own time. The MoCA required scheduling a videoconference session with the experimenter. Thus, not all participants who completed the ORCA-PD also completed the MoCA (56/112). This higher completion rate of the ORCA-PD compared to the MoCA is another indication of the higher utility of the ORCA-PD as a self-administered task. There were no significant differences in MoCA scores between the groups (*t*_26.87_=1.35; *P=.*19).

Importantly, we selected participants with PD who had relatively high cognitive scores, allowing us to test whether our new online cognitive assessment could distinguish between cognitively similar PD and NT groups. This is critical for developing a test that is sensitive to subtle cognitive group differences between participants with PD with early cognitive dysfunction and NT participants.

Participants with PD were primarily recruited through the Center for Accessible Neuropsychology (CAN) database, which includes individuals who had previously participated in CAN assessments or responded to online advertisements (eg, via PD associations such as the Israel Parkinson Association). NT participants were recruited online through the CAN database and through relatives or caregivers of participants with PD.

Inclusion criteria required participants to be aged between 40 and 90 years and fluent in Hebrew or English, with PD diagnosed after the age of 40 years, with varying degrees of PD stage (Hoehn and Yahr [H&Y] scale ≤4), and with or without MCI (19<MoCA<26). Individuals with other neurological (non-PD) or psychiatric disorders, learning disabilities, or significant visual or auditory impairments were excluded.

**Table 1.** Demographic and clinical characteristics for study participants (N=112).

Participant group	Participants, n	Age (y), mean (SD)	Education (y), mean (SD)	Women, n/N (%)	MoCA^a^, mean (SD)	MDS-UPDRS III^b^, mean (SD)	Disease duration (y), mean (SD)
Parkinson disease	56	67.2 (8.0)	17.5 (4.7)	28/56 (50.0)	25.2 (3.1)	21.9 (14.2)	7.4 (11.4)
Neurotypical controls	56	64.4 (9.4)	15.3 (3.3)	29/56 (51.8)	26.4 (2.9)	—^c^	—

a MoCA: Montreal Cognitive Assessment.

b MDS-UPDRS III: The International Parkinson and Movement Disorder Society—Unified Parkinson’s Disease Rating Scale Part III.

c Not applicable.

A total of 145 participants were enrolled in the study, of whom 135 (93%: English=91.5%, Hebrew=96.1%) met the inclusion criteria. Twelve participants were excluded based on eligibility criteria (2 with attention-deficit/hyperactivity disorder, 2 with dyslexia, and 8 who did not meet age requirements), and 1 participant was excluded due to incomplete data, leaving 122 eligible participants. In this sample, the youngest participant with PD was 46 years old. Accordingly, the control group was restricted to the same minimum age to ensure comparability between groups. From this pool, 10 control participants were randomly selected to obtain groups with comparable age distributions, resulting in a final sample of 112 participants (English=76 and Hebrew=36).

### Neurological and Neuropsychological Assessment

For a subgroup of participants, we administered neuropsychological testing. We followed the International Protocol for Online Neuropsychological Testing [5-7,38-40]. This protocol includes a script and procedure for participant recruitment through online platforms and for conducting online evaluations [6]. The experimenter contacted interested participants via email to schedule a live interview. Prior to each session, video and audio issues were addressed to minimize disruptions. If technical difficulties arose during the session, they were typically resolved within a few minutes. In rare cases (<5%, 5/112) where the technical difficulties could not be resolved, the session was rescheduled.

After completing the informed consent form, participants completed a demographic and medical questionnaire. Through the questionnaire, the experimenter collected medical history information, including age at diagnosis, medication, primary symptoms, genetic subtype, diet, other neurological or psychiatric conditions, and other relevant information pertaining to their health and lifestyle. Information regarding medications (eg, carbidopa or levodopa, cholinesterase inhibitors, and *N*-methyl-d-aspartate receptor antagonists) was collected through the medical history questionnaire. Because medication type, dosage, and timing varied across participants, and detailed pharmacological modeling was beyond the scope of this validation study, medication effects were not included in the analyses.

The experimenter then administered the MoCA (version 8.1) in the participant’s preferred language (Hebrew or English). The MoCA was administered via videoconference following the official guidelines on the MoCA website. Since the MoCA assessment was administered online, several adjustments were made based on previous papers [40-42]. Participants were instructed in advance and at the start of the session to have a pen and paper available. Visual items (eg, visuospatial and naming tasks) were presented via screen sharing. For the Trail Making Test, responses were provided verbally. For the drawing tests, participants were asked to draw at home and show the drawing on the screen to the experimenter. First, participants were shown a cube on the screen with the label “Copy cube” and were instructed to replicate it. Next, participants were presented with the label “Draw clock” and were asked to draw a clock with a specific time given by the experimenter. For Naming, stimuli were presented individually. For orientation, participants were asked to close their eyes and report the date and location. The MoCA took approximately 10 minutes to complete [18,40].

To assess the motor severity of participants with PD, the experimenter next administered the International Parkinson and Movement Disorder Society—Unified Parkinson’s Disease Rating Scale Part III (MDS-UPDRS III) as well as the H&Y scale [43]. Then, a link to the self-administered ORCA-PD test and the usability questionnaire was sent to participants’ emails to be completed within 2 weeks of the videoconferencing assessment. Participants completed ORCA-PD in the same language as the MoCA to ensure linguistic consistency.

### ORCA-PD: Remote, Unsupervised, and Self-Administered Cognitive Tool

**Figure 1.**
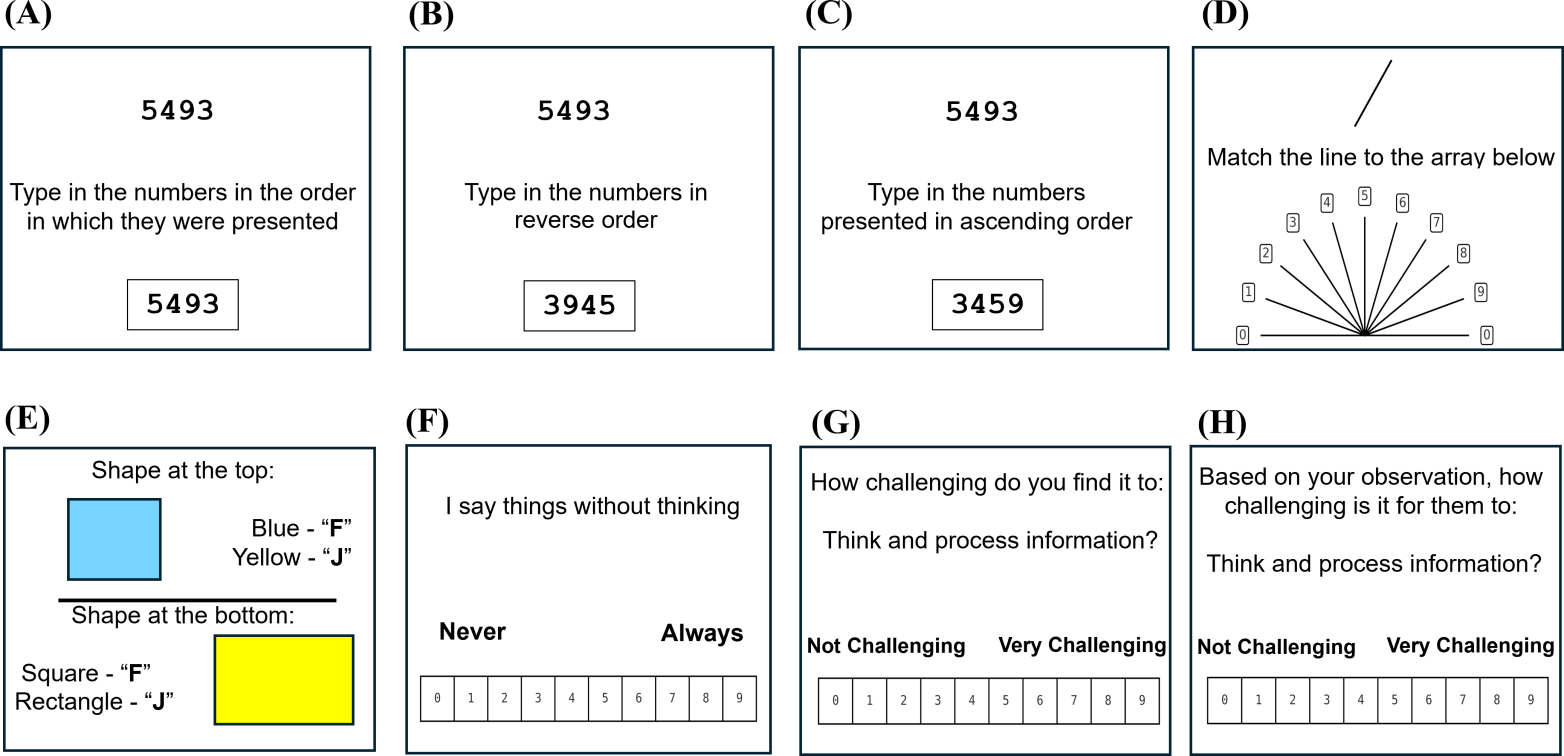
The measures of ORCA-PD (Online Rapid Cognitive Assessment for Parkinson’s Disease): (A) digit span forward, (B) digit span backward, (C) digit span order, (D) line orientation match, (E) alternate task switching, (F) impulsivity, (G) subjective cognitive complaints—self-assessment, and (H) subjective cognitive complaints—other assessment.

The ORCA-PD comprises 8 brief cognitive measures—5 performance-based tasks and 3 questionnaires—tailored to be sensitive to PD (Figure 1). These measures were selected based on converging evidence from meta-analyses and comprehensive reviews identifying cognitive measures that are sensitive to PD, yielding medium-to-large effect sizes when differentiating PD from NT participants [11-13,44,45]. Additionally, all tasks were adapted for online administration and optimized for older participants. For example, visual stimuli were designed to be large and simple, enhancing accessibility for older adults. To reduce the influence of PD-related motor impairments, motor demands were minimized in each task. For example, instead of using a task that required precise mouse control, which could potentially affect cognitive measurement in PD as a motor impairment-related confounder, the tasks were mostly implemented using simple forced 2-choice response paradigms. In addition, accuracy was emphasized as the primary dependent variable.

The first 3 tasks in the ORCA-PD were 3 versions of the digit span (DS) task. These consisted of digit span forward (DSF) for establishing baseline abilities, digit span backward (DSB) [11] (effect size=0.46), and digit span order (DSO) [12] (effect size=0.75). Each DS task consisted of 2 difficulty levels, with level 2 (hard) comprising longer digit sequences than level 1 (easy). Sequence lengths for level 1 were 4 (DSF and DSB) or 5 (DSO) and for level 2 were 6 (DSF and DSB) and 7 (DSO). These were selected based on previous work by Grogan et al [46]. Following recommendations from the Movement Disorder Society (MDS) Task Force [32], we adapted the Benton Judgment of Line Orientation task [47] for the fourth task, herein referred to as the line orientation match (LOM) [13] (effect size=0.52). Participants matched pairs of lines based on their orientation (9 options). For the LOM task, difficulty was defined by orientation discriminability, where trials with straight angles (0°, 180°, 90°) were classified as easy, while all other angles (18°, 36°, 54°, 72°, 108°, 126°, 144°, and 162°) were classified as hard. Each orientation was presented twice, resulting in 4 easy trials and 16 hard trials. The fifth cognitive task was an alternate task switching (ATS) task involving shape and color categorization, based on target location, consistent with MDS recommendations and prior meta-analyses [11,32,48]. To reduce motor demands [5], the participant responded by pressing a single key per trial (effect sizes for similar paradigms, such as the Trail Making Test B, were 0.67 to 0.89). Trials were categorized as repeat (same as trial n−1; easy) or switch (different from trial n−1; hard), enabling assessment of task sensitivity. The task comprised 33 trials: 16 repeat trials, 16 switch trials, and 1 initial trial.

The sixth measure was a short questionnaire assessing impulsivity [49]. Based on a recent meta-analysis [44], we used items from the Barratt Impulsiveness Scale (BIS-11). Specifically, we selected items only from the BIS-11 subscales that were found to be sensitive to PD [44] (ie, attention and self-control). From these 2 nonmotor subscales, we selected 7 items that were found to be the most sensitive to PD [50], with responses scored on a scale of 0 to 9. These items represent a targeted subset of the BIS-11 and do not constitute a stand-alone validated impulsivity scale. The final 2 measures assessed subjective cognitive complaints (SCC) [51] using both self-report and informant-report questionnaires. The inclusion of SCC was motivated by prior evidence indicating that SCC are present in approximately 36% of individuals with PD [45]. The questionnaires assessed 6 domains: global cognition and 5 specific cognitive domains (attention, memory, visuospatial function, executive function, and language), consistent with domains recommended by the MDS Task Force [52]. Informant ratings were obtained from individuals familiar with the participant’s everyday functioning (eg, spouse, partner, caregiver, or close friend). Responses were scored on a scale of 0 to 9 and normalized to a range of 0 to 1.

Each of the eight measures was scored from 0 to 1, with higher values reflecting better performance. To standardize the questionnaire scores from 0 to 1, each score was adjusted so that higher scores would indicate less impairment by reversing the score where necessary. Accordingly, a greater impulsivity score indicated lower impulsivity, and a higher SCC score indicated lower concern. The total score for each questionnaire was averaged and then divided by 9 to yield a scale ranging from 0 to 1. Finally, the total ORCA-PD score was calculated as the simple mean of the 8 measures for each participant, such that each task and questionnaire contributed equally to the final score (ORCA-PD naive score).

Upon completing the ORCA-PD, participants were asked to provide feedback using the usability questionnaire comprising 4 questions, each rated on a 5-point Likert scale.

Although partial data could be recovered in cases of interruption, the predefined criterion for analysis was the completion of the full assessment. In cases where a session was interrupted, participants were typically provided with a new access link and asked to restart the assessment. When multiple attempts occurred, only the fully completed session was included in the analysis. In practice, interruptions were uncommon (<12% of sessions), and cases involving mid-test disconnections with incomplete data were rare (<2%).

### Statistical Analysis

Data preprocessing and visualization were performed in R (version 4.3.2; RStudio Team, 2023) [53]. The correlation heatmap was generated in Python. To evaluate group differences (eg, NT vs PD), independent samples *t* tests were used for continuous variables (eg, ORCA-PD score), and chi-square tests were used for categorical variables (eg, gender). These comparisons were evaluated using 2-tailed tests. For hypothesis-driven analyses of ORCA-PD outcomes, group comparisons were evaluated using 1-tailed tests when the direction of the effect was specified a priori. Where appropriate, group comparisons were evaluated using linear regression, adjusting for years of education and age as covariates. Correlations between measures were assessed using Pearson correlation coefficient *r* or Spearman rank correlation ρ, depending on variable distribution and scaling. Convergent validity was assessed via correlations with MoCA scores, where it was hypothesized that ORCA-PD scores would be positively associated with MoCA scores. Construct validity was assessed via group comparisons (NT vs PD; MCI vs non-MCI). It was hypothesized that the MCI group would demonstrate lower ORCA-PD scores than the group without MCI, and that the PD group would demonstrate higher ORCA-PD scores than the NT group.

The internal structure of task-based measures was evaluated by examining whether participants’ performance declined with increasing task difficulty. Internal consistency of questionnaire-based measures was assessed using Cronbach α and McDonald ω. In addition, a correlation matrix of all ORCA-PD components was computed to further characterize the internal structure of the ORCA-PD measures. Discriminant validity was also assessed via the association between the ORCA-PD and the MDS-UPDRS III scores. As a secondary exploratory analysis, potential language-related effects were examined by comparing performance between English- and Hebrew-speaking cohorts within each group.

### Ethical Considerations

This protocol was approved by the Tel Aviv University Institutional Review Board Committee (#0005713‐6). All participants provided informed consent.

## Results

**Figure 2.**
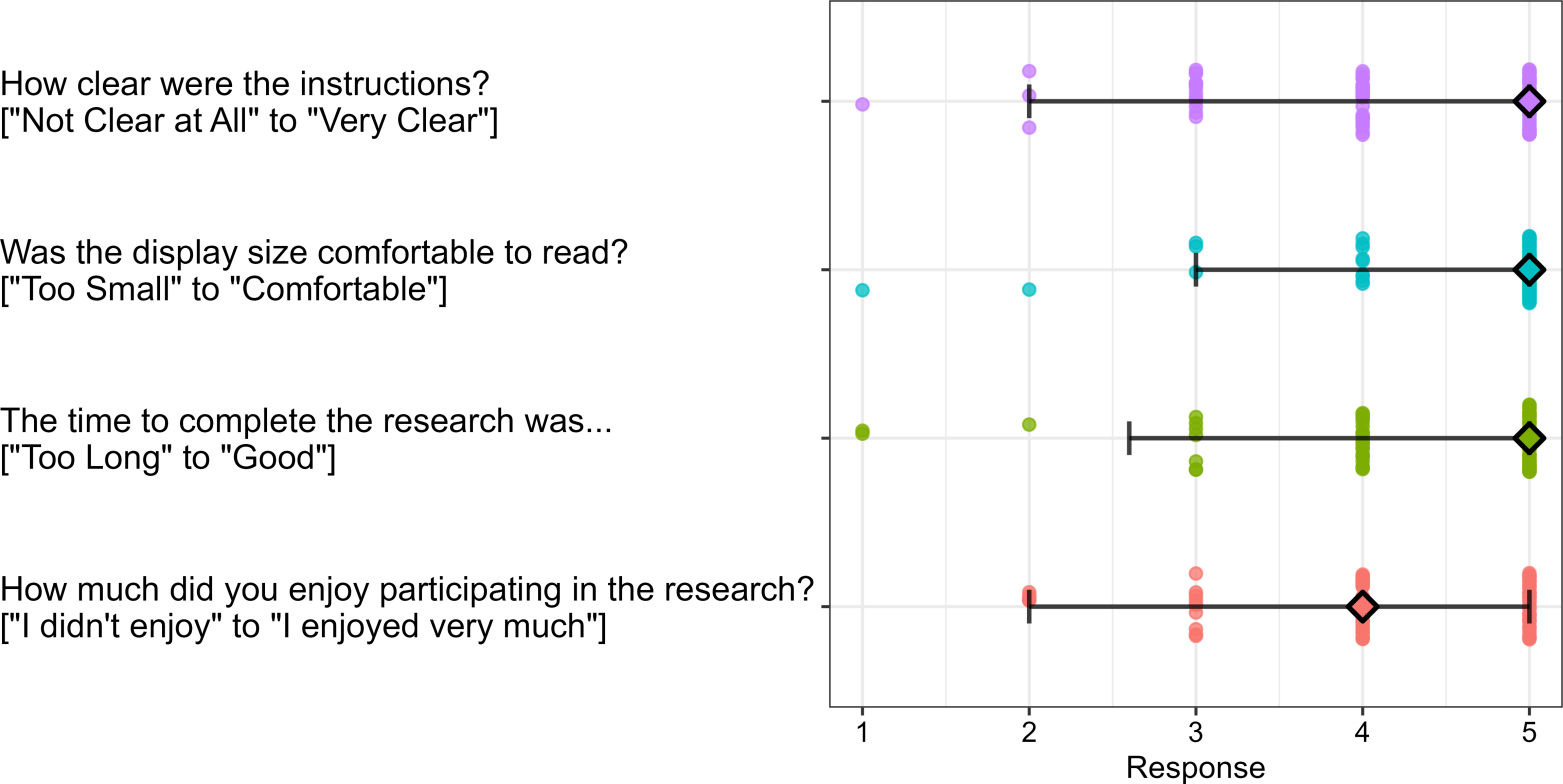
Participant (N=112) responses to the usability questionnaire after completing the ORCA-PD (Online Rapid Cognitive Assessment for Parkinson’s Disease). Four questions addressed the clarity of questions, font size, completion time, and overall enjoyment of participation. The median is marked with a diamond symbol.

On average, participants completed the ORCA-PD in 15 (SD 4.5; range 12‐25) minutes. This reflects task completion time only and does not include presession setup (eg, audio or video checks) or any additional interaction time. Even with this distinction, this reflects minimal participant burden and supports the ORCA-PD’s usability for remote assessment. Participant feedback further supported usability: median ratings indicated that instructions were very clear (5, IQR=1), the display size was comfortable (5, IQR=0), the overall experience was enjoyable (4, IQR=1), and the completion time was good (5, IQR=1) (Figure 2).

**Figure 3.**
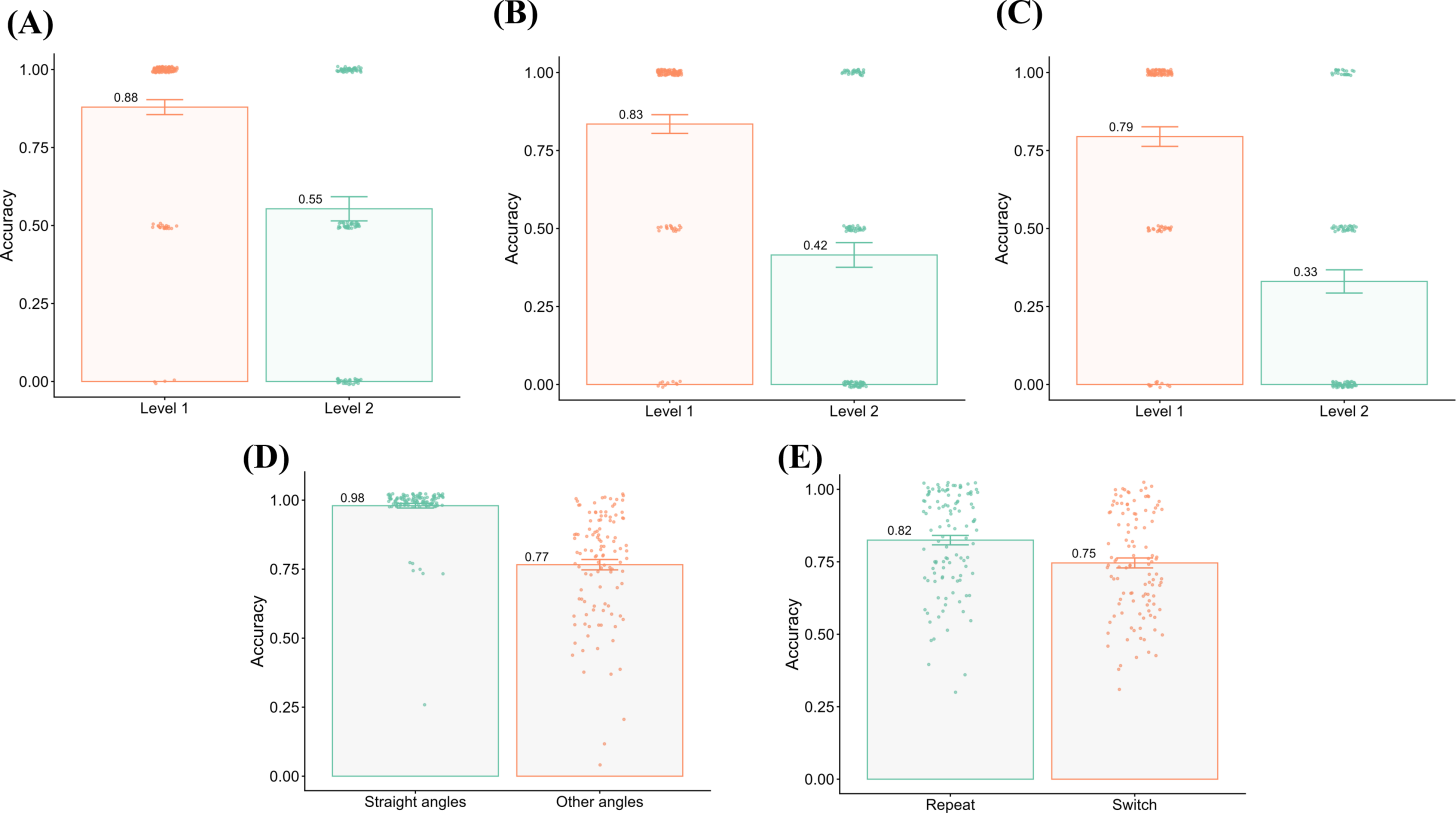
Participant (N=112) accuracy as a function of difficulty level for all ORCA-PD (Online Rapid Cognitive Assessment for Parkinson’s Disease) tasks. Accuracy decreased as task difficulty increased in each of the 5 tasks, supporting the expected performance pattern of the ORCA-PD online measures. (A) Digit span forward, (B) digit span backward, (C) digit span order, (D) line orientation match, and (E) alternate task switching. Each data point represents a single participant. Bars represent group means. The error bars represent SEs.

To assess the ORCA-PD’s sensitivity to expected patterns of task difficulty, we evaluated whether participant performance declined with increasing task difficulty. Such a pattern would support the expected performance pattern (ie, sanity check) of the online tasks in measuring the intended cognitive constructs (Figure 3). As expected, in 3 versions of the digit span task, participants performed significantly better on the easy level (level 1) than on the hard level (level 2) trials (forward: *t*_111_=8.99, *P*<.001; Cohen d=0.85; backward: *t*_110_=10.20, *P*<.001; Cohen d=0.97; order: *t*_111_=11.1, *P*<.001; Cohen d=1.05), supporting the expected performance pattern of these online tasks. In the LOM task, participants performed significantly better on the easy trials (*t*_111_=12.18, *P*<.001; Cohen d=1.15). In addition, easy trials showed high accuracy (98%), whereas the hard trials showed lower mean accuracy (76.6%). In the ATS, participants performed significantly better on repeat (easy) trials than on switch (hard) trials (*t*_111_=6.23, *P*<.001; Cohen d=0.59).

Regarding questionnaires, for impulsivity, internal consistency was medium (Cronbach α=0.68; McDonald ω=0.80). For the self-reported SCC questionnaire, internal consistency was good (Cronbach α=0.83; McDonald ω=0.91). For the informant-reported SCC questionnaire, internal consistency was strong (Cronbach α=0.90; McDonald ω=0.92).

**Figure 4.**
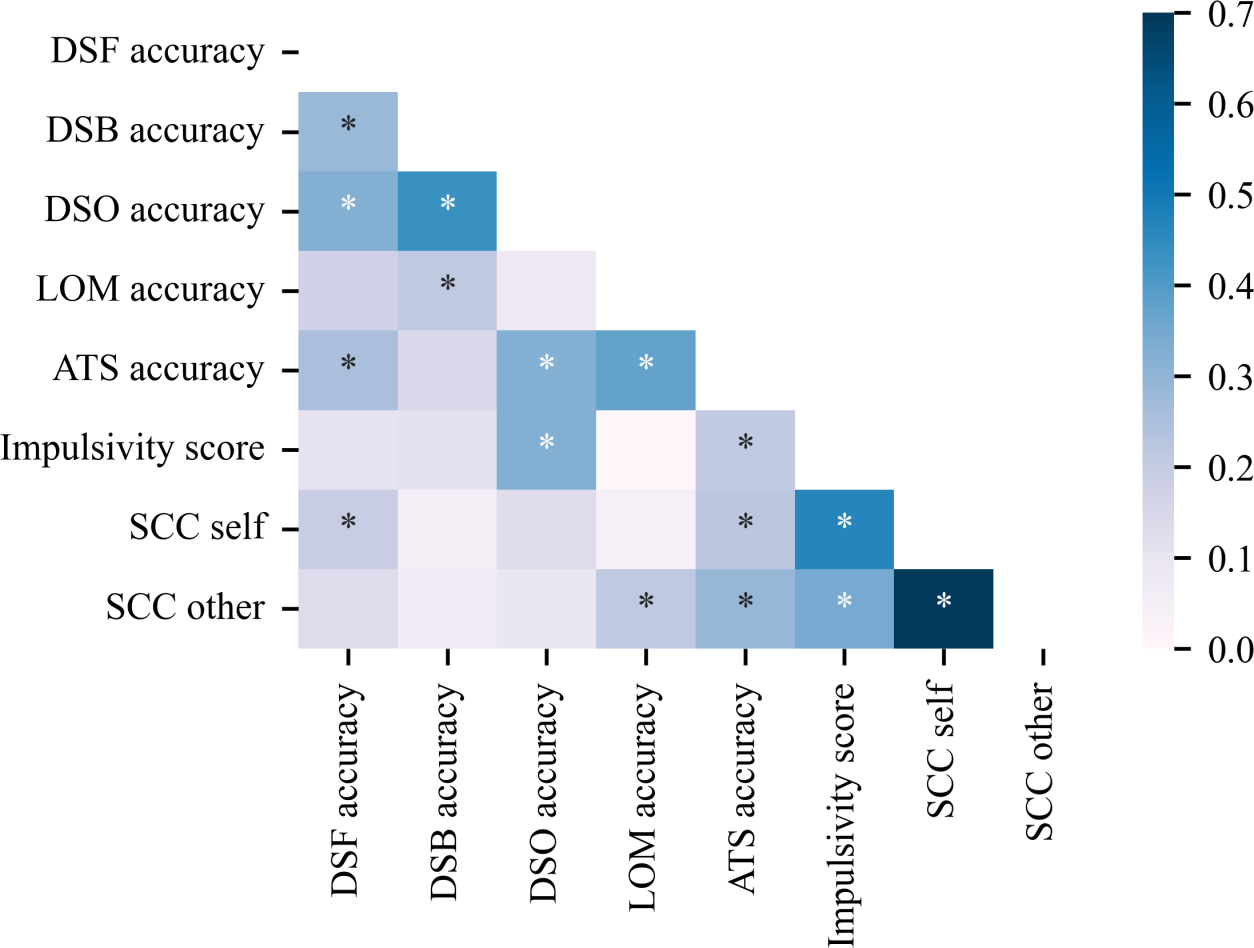
Correlation heatmap of the ORCA-PD (Online Rapid Cognitive Assessment for Parkinson’s Disease) task components (N=112). Pearson correlations are displayed using a blue color scale, where deeper shades represent stronger relationships. * indicates *P*<.05. ATS: alternate task switching; DSB: digit span backward; DSF: digit span forward; DSO: digit span order; LOM: line orientation match; SCC: subjective cognitive complaints.

To examine relationships among the different ORCA-PD measures, we computed a correlation matrix of all ORCA-PD components across participants (Figure 4). The heatmap below displays interfeature correlations (range –0.04 to 0.70), which is consistent with the multidimensional structure of the ORCA-PD.

Additionally, to evaluate the discriminant validity of ORCA-PD and its ability to dissociate cognitive from motor functions, correlations were examined with a motor measure, the MDS-UPDRS III. As expected, no significant correlations were found between any ORCA-PD measures and the MDS-UPDRS III scores, supporting the notion that ORCA-PD primarily assesses cognitive abilities (DSF: ρ=–0.25, *P*=.11; DSB: ρ=–0.19, *P*=.25; DSO: ρ=–0.24, *P*=.14; ATS: ρ=–0.07, *P*=.67; and LOM: ρ=–0.09, *P*=.57).

**Figure 5.**
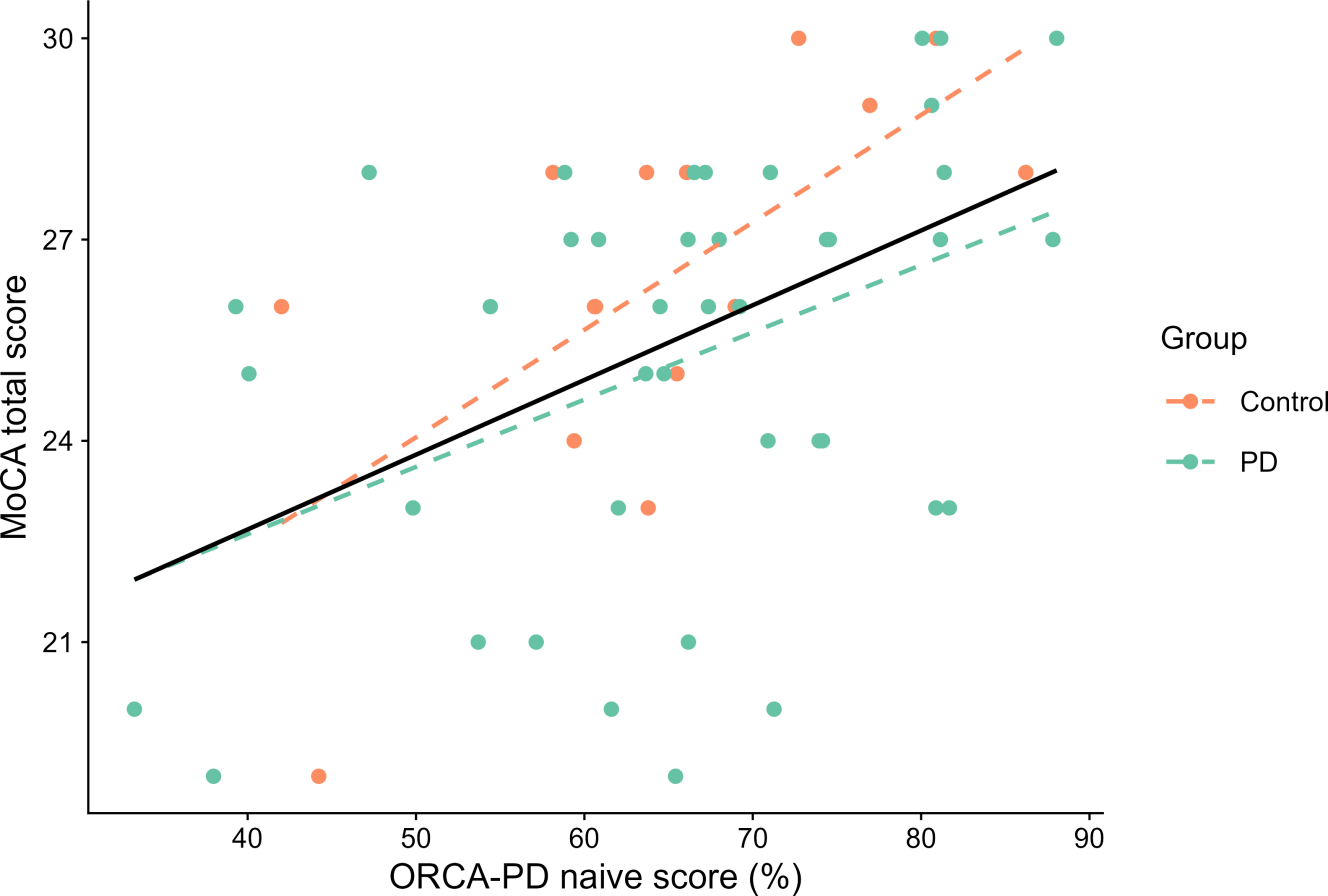
Scatterplot showing the relationship between ORCA-PD naive scores and MoCA scores (n=56). Dashed lines indicate group-specific trends, while the solid black line indicates the overall trend across both groups. MoCA: Montreal Cognitive Assessment; ORCA-PD: Online Rapid Cognitive Assessment for Parkinson’s Disease; PD: Parkinson disease.

Importantly, to assess convergent validity, we examined the correlation between the ORCA-PD naive score and MoCA scores (Figure 5, n=56). As expected, across both groups, a significant positive correlation was observed (ρ=0.45, 95% CI 0.21-0.64; *P*<.001), indicating a moderate association. When examined separately, significant associations were observed in both the PD (ρ=0.41, 95% CI 0.12-0.64; *P*=.004) and NT (ρ=0.62, 95% CI 0.15-0.86; *P*=.007) groups, supporting the preliminary validity of ORCA-PD.

To examine the ability of ORCA-PD to distinguish between people with or without MCI, we classified participants according to established criteria for MCI (ie, MoCA between 19 and 25) [54]. In a linear regression analysis adjusting for education and age, neither education (β=0.37; *P=.*34) nor age (β=–0.27; *P=.*18) was a significant predictor of ORCA-PD naive scores. As expected, the non-MCI group demonstrated higher ORCA-PD naive scores compared to the MCI group (β=6.07; *P=.*046). When restricting the analysis to PD only, neither education (β=0.37; *P=.*40) nor age (β=–0.23; *P=.*40) was a significant predictor of ORCA-PD naive scores. As expected, the non-MCI group again demonstrated higher ORCA-PD naive scores compared to the MCI group (β=7.22; *P=.*046). This analysis was not conducted on NT alone, as there were fewer than 5 participants in this group with MCI. These results support the discriminative ability of the ORCA-PD, demonstrating that it can capture cognitive impairments.

**Figure 6.**
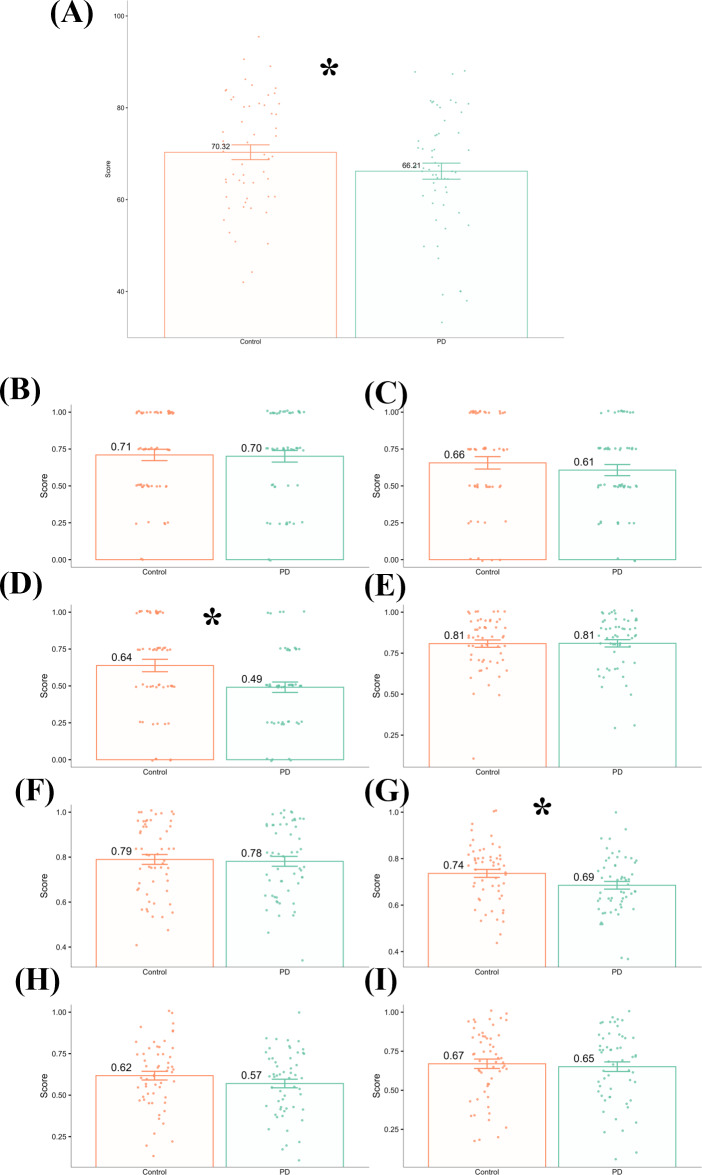
Comparison between Parkinson disease (PD) and controls (N=112) for (A) ORCA-PD (Online Rapid Cognitive Assessment for Parkinson’s Disease) naive score, (B) digit span forward accuracy, (C) digit span backward accuracy, (D) digit span order accuracy, (E) line orientation match accuracy, (F) alternate task switching accuracy, (G) impulsivity score, (H) subjective cognitive complaints—self-assessment, and (I) subjective cognitive complaints—other assessment. * indicates *P*<.05.

Importantly, the ORCA-PD’s discriminative ability was also assessed by comparing ORCA-PD scores between PD and NT (Figure 6). In the regression model, neither education (β=0.27; *P=.*36) nor age (β=–0.19; *P=.*16) was a significant predictor of ORCA-PD naive scores. As expected, the group effect was significant (β=–4.18; *P=.*048), with PD scoring lower than NT.

To explore the specific cognitive domains contributing to the group differences, the NT and PD groups on all individual measures were compared. NT participants performed significantly better than PD on DSO accuracy (*t*_107_=2.67, *P*=.004). In addition, NT participants scored significantly higher in impulsivity than participants with PD (*t*_110_=2.15, *P*=.02). No significant group differences were found on DSF (*t*_110_=0.16, *P*=.44), DSB (*t*_109_=0.87, *P*=.19), ATS (*t*_110_=0.27, *P*=.39), LOM (*t*_110_=–0.06, *P*=.52), or SCC measures (self: *t*_110_=1.28, *P*=.10 and other: *t*_110_=0.44, *P*=.33).

As an exploratory analysis, which needs to be further validated in future studies, we compared scores between English- and Hebrew-speaking cohorts. At the level of overall ORCA-PD naive scores, differences between English- and Hebrew-speaking participants were not statistically significant in the NT group (*t*_27.49_=2.02, *P*=.05). However, in the PD group, the English-speaking cohort had significantly higher scores (*t*_31.67_=2.19, *P*=.04). At the task level, language-related differences were observed for digit span forward in the NT group, where performance was significantly higher in the English-speaking cohort (*t*_20.16_=2.90, *P*=.009). In the ATS task in the PD group, performance was higher in the English-speaking cohort (*t*_36.49_=2.19, *P*=.04). For all other measures, no significant language differences were observed in the NT group (DSB: *P*=.68; DSO: *P*=.19; LOM: *P*=.29; ATS: *P*=.17; impulsivity: *P*=.29; SCC-self: *P*=.79; SCC-other: *P*=.96) or in the PD group (DSF: *P*=.05; DSB: *P*=.92; DSO: *P*=.16; LOM: *P*=.72; impulsivity: *P*=.19; SCC-self: *P*=.06; SCC-other: *P*=.08).

## Discussion

The current in-person cognitive screening tools face challenges related to accessibility, scalability, and PD specificity. We aimed to develop and tailor a new sensitive tool capable of identifying cognitive impairments in early- to mid-stage PD in a manner that was both accessible and scalable. Accordingly, ORCA-PD was developed as a fully self-administered, web-based cognitive screening tool tailored to capture PD-specific cognitive impairments.

First, as reflected by high completion rates (93%) and participant ratings (4-5 out of 5), ORCA-PD showed high usability, further supporting its feasibility across diverse populations (more than 30 geographical locations). Second, ORCA-PD naive scores correlated with MoCA scores, supporting preliminary validity. While the NT estimate should be interpreted with caution due to the small sample size, the moderate correlation with MoCA suggests that ORCA-PD captures overlapping but not identical constructs, offering information beyond a generic cognitive screener. As with all screening tools designed to enable accessible and fast identification of cognitive impairment, ORCA-PD was not meant to replace comprehensive assessment tools but to prompt early diagnosis of PD with MCI. The moderate correlation with the MoCA supports ORCA-PD’s role as a complementary tool, particularly when subtle, domain-specific changes are expected earlier in the PD course [52]. Third, ORCA-PD differentiated between groups, with performance following expected trends: NT>PD and PD-non-MCI>PD-MCI, confirming its discriminative capacity.

When examining previous literature, recent studies have begun to explore the potential of remote cognitive testing in PD. One study [28] demonstrated the feasibility and test-retest reliability of an unsupervised online cognitive battery in PD. However, the study did not assess validity, nor did it evaluate MCI using an additional screening measure. Another study [31] examined smartphone-based cognitive testing in daily contexts, but the sample size was small (n=27) and required a supervised laboratory session. A third study [27] demonstrated the feasibility of large-scale remote data collection in participants with PD using self-report questionnaires, but it lacked performance-based cognitive measures. A fourth pilot study [29] consisted of virtual neuropsychological testing using traditional batteries with a small sample size (n=35) and reliance on supervised administration. A fifth study [30] evaluated a lengthy 19-task online cognitive battery in individuals with PD and individuals with REM (rapid eye movement) sleep behavior disorder, identifying a post hoc 20-minute subset sensitive to group differences. However, the tasks relied heavily on reaction time, including motor-dependent tasks (ie, mouse use), which can confound cognitive measurement in PD. Additionally, the tasks were not chosen based on previous empirical independent findings and were not prospectively tested. In contrast, ORCA-PD was prospectively evaluated. The task selection was guided by prior meta-analyses and reviews that provided independent evidence for their sensitivity to PD. Additionally, the ORCA-PD tasks were specifically designed to reduce reliance on motor skills, thereby reducing the risk of motor-related confounds. This was also demonstrated by the lack of correlation between the ORCA-PD measures and the MDS-UPDRS III scores.

We developed and tailored ORCA-PD as a cognitive assessment for early- to mid-stage PD. In our sample, although inclusion criteria allowed participants with H&Y scores of 4 or lower, 98% had an H&Y score of 3 or lower (none had a score of 5; mean MDS-UPDRS III=21.9, mean disease duration=7.4 y). These findings are consistent with mild-to-moderate motor severity [55].

Of note, ORCA-PD was designed for remote, unsupervised administration, which likely contributed to the high rates of completion and usability observed, supporting its potential clinical applicability. Our sample did not include participants with dementia, as ORCA-PD was designed for early- to mid-stage PD, and self-administered testing may be less feasible in advanced stages. Taken together, validating ORCA-PD in this disease stage is advantageous because it targets the period when mild cognitive changes emerge.

Our study has several limitations. First, some measures (eg, SCC) showed limited sensitivity to between-group differences. Future studies should refine these tasks or replace them with more PD-sensitive alternatives, such as language-based measures. Second, although we assessed validity, we did not evaluate test-retest reliability. Third, the scoring of some tasks (eg, digit span) is relatively coarse, where a small number of trials per difficulty level may limit granularity. Yet, the ORCA-PD design is typical of screening instruments, such as the MoCA and MMSE. The ORCA-PD, in fact, includes more trials than these widely used measures (4 vs 2 in digit span), which may provide greater sensitivity for detecting impairments. Our primary goal at this stage was to implement a simple approach that allows direct comparison across predefined difficulty levels, serving as an assessment of the expected performance pattern (ie, sanity check) of the online task design. In addition, we prioritized accuracy-based scoring over reaction time to minimize the influence of motor slowing, which may disproportionately affect reaction time measures in PD. While reaction time may provide additional sensitivity, it may also introduce motor confounds. Fourth, MoCA data were available for only a subset of participants, resulting in a smaller sample. Although this subsample exceeded the minimum sample size required by the power analysis, the small sample size may limit the generalizability of these findings. Fifth, the MoCA was used both as a measure for convergent validity and to operationalize MCI classification, introducing a degree of circularity that limits the independence of the validation and may inflate observed associations. Sixth, medication status at the time of testing was not controlled. A majority of participants (<70%) were treated with dopaminergic medication, and the median time since the last medication intake was 120 minutes. Although prior work [56,57] suggests that dopaminergic medication has limited effects on cognitive screening measures such as the MoCA, with more modest and domain-specific effects, variability in medication state may have introduced additional heterogeneity in performance. This study was not designed to examine ON versus OFF medication effects, and future studies should systematically evaluate the influence of medication state and timing of testing on ORCA-PD performance.

Seventh, ORCA-PD scores were not compared against a comprehensive neuropsychological battery, and the use of a single-instrument cutoff does not meet MDS level II criteria. Future research should validate ORCA-PD against independent, gold-standard neuropsychological assessments and include parallel forms to estimate test-retest reliability, while recognizing that ORCA-PD is designed primarily as a screening tool. Eighth, online-based participation may favor individuals who are more technologically adept or less cognitively impaired; therefore, future studies should directly compare online and in-person administrations to assess potential selection and usability biases.

Ninth, some NT participants were recruited through relatives or caregivers of individuals with PD, which may introduce selection bias. Individuals in close proximity to people with a PD diagnosis may differ from the general population in terms of health care engagement and motivation to participate in research. This may limit the external validity of the findings, as the control group may not be fully representative of the general population.

In addition, race, quality of education, area deprivation index, and related factors were not collected. This represents a substantive limitation, particularly given the goal of developing an accessible tool for diverse populations, and it restricts our ability to evaluate generalizability across demographic and socioeconomic contexts. This is an important goal for future studies.

Language-related effects were examined as a secondary exploratory analysis. While some differences were observed (eg, DSF), these effects were not consistent across tasks or groups. However, our study was not designed or powered to evaluate cross-language differences or establish language-specific norms. Additionally, we did not aim to validate ORCA-PD in different languages. The relatively small sample, along with important potential demographic and cultural differences between language groups, further limits interpretability. Future studies should be designed to establish language-specific norms and examine between-language differences. Finally, the cross-sectional design precluded longitudinal inferences, and future longitudinal studies are needed to examine ORCA-PD’s sensitivity to cognitive change over time. Addressing these limitations will help maximize the tool’s clinical and research utility.

Although we developed ORCA-PD for people with PD, ORCA’s framework and principles can be expanded. Additional studies could focus on different populations, with an emphasis on varying levels of education and both rural and urban regions. Given the relatively high education level of our current sample, future validation in less-educated and lower-resource populations will be critical to ensure accessibility and generalizability. Socioeconomic information was not collected in this study, which limits our ability to assess the representativeness of our sample across social classes; future work should incorporate these measures to evaluate equity of access and usability for all. Furthermore, this platform need not be limited to PD. By selecting tasks matched to PD cognitive signatures and adjusting task difficulty and response modalities, our ORCA approach can be adapted to other conditions (eg, cerebellar ataxia). Such adaptations will require independent validation and condition-specific calibrations.

In summary, ORCA-PD is a brief, self-administered cognitive screening test for people with PD that demonstrates feasibility, utility, and preliminary validity that can be used in research and clinical settings upon further validation. By enabling unsupervised home testing, it has the potential to complement clinic-based screening and support between-visit cognitive monitoring. ORCA-PD could provide an accessible, scalable, and specific tool for cognitive screening in PD.

## Abbreviations

ATS: alternate task switching
BIS-11: Barratt Impulsiveness Scale
CAN: Center for Accessible Neuropsychology
DSB: digit span backward
DSF: digit span forward
DSO: digit span order
H&Y: Hoehn and Yahr scale
LOM: line orientation match
MCI: mild cognitive impairment
MDS: Movement Disorder Society
MDS-UPDRS III: Movement Disorder Society—Unified Parkinson’s Disease Rating Scale, Part III
MMSE: Mini-Mental State Examination
MoCA: Montreal Cognitive Assessment
NT: neurotypical controls
ORCA-PD: Online Rapid Cognitive Assessment for Parkinson’s Disease
PD: Parkinson disease
SCC: subjective cognitive complaints
